# Detection of t(14;16)(q32;q22) and Monosomy 13 by FISH Analysis in a Patient with Multiple Myeloma Associated with Sjögren's Syndrome: The First Case Report from India

**DOI:** 10.1155/2013/279801

**Published:** 2013-01-16

**Authors:** Rupesh R. Sanap, Arundhati S. Athalye, Prochi F. Madon, Boman N. Dhabhar, Mahendra B. Sute, Amit A. Mahabale, Dhanashree J. Warang, Firuza R. Parikh

**Affiliations:** ^1^Department of Assisted Reproduction and Genetics, Jaslok Hospital and Research Centre, 15 Dr. G. Deshmukh Marg, Mumbai 400026, India; ^2^Department of Oncology, Jaslok Hospital and Research Centre, 15 Dr. G. Deshmukh Marg, Mumbai 400026, India

## Abstract

Sjögren's syndrome (SS) is a chronic slowly progressive autoimmune disorder characterized by symptoms of oral and ocular dryness, exocrine dysfunction, and lymphocytic infiltration of exocrine glands. Multiple myeloma (MM) is a bone-marrow-based malignant neoplasm of plasma cells associated with serum/urine monoclonal paraproteins and lytic skeletal lesions. There have been very few reported cases of MM, who had SS as the first presentation. We report a case of a woman diagnosed with Sjögren's syndrome, who was later suspected to have multiple myeloma on serum protein electrophoresis. Fluorescence *in situ* hybridization (FISH) was carried out to check for deletions of loci 13q14.3, ATM, p53, and IGH (14q32) rearrangements on a bone marrow aspirate. Monosomy 13 was observed in 49% of cells, and a rearrangement at the IGH locus was seen in 42% of cells. To determine the partner chromosome associated with the IGH rearrangement, further FISH tests were set up for t(4;14)(p16;q32) followed by t(14;16)(q32;q22) on fresh slides. The test was negative for t(4;14) but positive for t(14;16) in 27% of cells. This confirmed the diagnosis of MM. We report the first case from India, having an association of Sjögren's syndrome with multiple myeloma, which showed t(14;16) and monosomy 13 by FISH analysis.

## 1. Introduction

Sjögren's syndrome (SS) is a chronic slowly progressive autoimmune disorder characterized by symptoms of oral and ocular dryness, exocrine dysfunction and lymphocytic infiltration of exocrine glands [1]. SS is predominantly the disease of middle-aged women, while myeloma is a disease of the elderly, with only 2% of cases occurring in patients <40 years of age. 

Multiple myeloma (MM) is a cancer of the plasma cells which comprise 5% of the cells in bone marrow (BM). In a MM patient, this number can double, causing very serious health problems. MM is a bone-marrow-based malignant neoplasm associated with serum and/or urine monoclonal paraproteins and lytic skeletal lesions [2]. It accounts for around ten percent of all hematologic malignancies [3]. Myeloma cells are typically CD56, CD38, and CD138 positive and CD19 and CD45 negative. Previous studies using metaphase cytogenetics reported often complex numerical and structural chromosome abnormalities in 30%–40% of patients with MM [4]. The use of DNA specific probes and the technique of FISH enables us to study chromosomal abnormalities in interphase nuclei [5].

There have been very few reported cases of MM, which had SS as the first presentation [6–15]. To date, there is only 1 case report from India of a patient with SS and MM [16], which was not subjected to cytogenetic analysis to check for chromosomal abnormalities present in MM.

## 2. Case Report

A suspected case of MM was referred to us for chromosomal analysis. The female patient, aged 62, had a history of dry mouth since 2 years, significant weight loss (82 kg to 65 kg with a BMI of 33.8) in 6 months, excessive dry cough with bleeding, a pneumonia patch on X-ray, dry eyes, no tears, and loss of appetite. The total lymphocyte count was 4900/cu mm, RBC 3.11 mill/mm^3^, erythrocyte sedimentation rate 100 mm at 1st hour and 160 mm at 2nd hour, Hb 8.9 gm/dL, ANA 1 : 100 (weak positive), and RA factor ++. Multiple patchy areas of ground glass opacities in the subpleural region of apical/basal segments of both lower lobes, lingula, right middle lobe, and anterior segment of right upper lobe were seen. USG showed gall stones. Creatinine and SGPT were normal. The patient was diagnosed with Sjögren's syndrome. She was on methylprednisolone, vitamins, and minerals.

Serum protein electrophoresis after 6 months showed total protein 10.9 gm/dL, globulin 9.16 gm/dL, hypoalbuminemia with decreased *β*2 region, gamma globulin 6.94 gm/dL, A/G ratio 0.36, and presence of M band in the gamma region (4.94 gm/dL). Hence multiple myeloma was suspected and the patient was referred to our laboratory for cytogenetic analysis.

**Figure 1:**
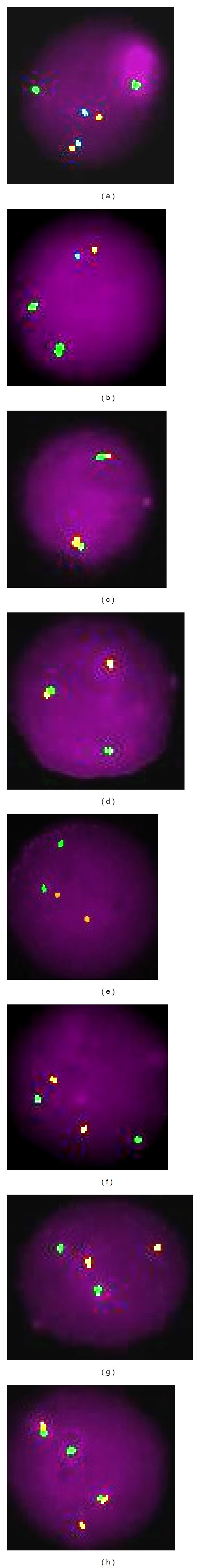
FISH images of normal and abnormal cells using various probes. (a) A normal cell showing 2 green (G), 2 orange (O) and 2 aqua (A) signals for chromosome 12 and loci 13q14.3 and 13q34 on chromosome 13, respectively, using Vysis CLL probe set for CEP12, 13q14.3 and 13q34. (b) A cell showing 2G1O1A signal pattern indicating monosomy 13 using Vysis CLL probe set for CEP12, 13q14.3, and 13q34. (c) Normal cell showing 2 fusion (F) signals for IGH locus (14q32) using Vysis IGH break-apart rearrangement probe. (d) A cell showing 1G1O1F signal pattern indicating IGH rearrangement at 14q32 using Vysis IGH break-apart rearrangement probe. (e) A normal cell showing 2 green and 2 orange signals for chromosomes 11 and 17, respectively, using Vysis CLL probe set for 11q23 (ATM) and 17p13.1 (p53). (f) A normal cell showing 2 orange and 2 green signals for chromosomes 4 and 14, respectively, using Vysis LSI IGH-FGFR3 dual colour dual fusion translocation probe. (g) A normal cell showing 2 green and 2 orange signals for chromosomes 14 and 16, respectively, using Vysis LSI IGH-MAF dual colour dual fusion translocation probe. (h) A cell showing 1G1O2F signal pattern indicating IGH-MAF fusion for t(14;16) using Vysis LSI IGH-MAF dual colour dual fusion translocation probe.

FISH was set up on the whole bone marrow sample using Abbott (Vysis) CLL FISH panel with probes for loci 13q14.3, 13q34 (control), ATM, p53, and CEP 12 [17]. The IGH break-apart probe to check for rearrangements at the IGH locus (14q32) was used initially. The FISH results mainly showed monosomy 13 in 49% and rearrangement at the IGH locus in 42% cells. Subsequently, the patient was tested for t(4;14)(p16;q32) which was negative. A further test was carried out to check for rearrangement of IGH with MAF (16q22-23). This showed the translocation t(14;16)(q32;q22) in 27% cells (Figure 1).

## 3. Discussion

Based on the revised international classification criteria for SS [18], this patient satisfied the diagnostic criteria of SS. Serum electrophoresis showed the presence of M band and more than 10% plasma cells on bone marrow aspiration. The diagnosis of MM was further supported by the presence of t(14;16) and monosomy 13 by FISH on BM. Monosomy 13 was one of the first recurrent chromosomal abnormalities found in MM [19]. Up to one-third of MM may emerge from preexisting monoclonal gammopathy of undetermined significance (MGUS), and monosomy 13 has been correlated with the transformation of MGUS to overt MM [20]. The cryptic translocation t(14;16)(q32;q22) was first detected by multicolour spectral karyotyping (SKY) as a nonrandom abnormality in MM [21].

According to the current risk stratification criteria for MM, t(14;16), t(14;20) and deletion 17p are stratified as high risk myeloma, whereas monosomy 13 or deletion 13, t(4;14) and hypodiploidy are considered to have intermediate risk disease [18]. All others are considered to have standard risk myeloma [3].

Our patient is currently on Bortezomib and is responding well. Her recent Hb is 10.5 gm/dL and her weight has increased to 70 kg. This is the first case report from India, describing an association of Sjögren's syndrome with multiple myeloma showing t(14;16)(q32;q22) and deletion 13q14.3 by FISH analysis.
